# Reduced Operating Time but Not Blood Loss With Cruciate Retaining Total Knee Arthroplasty

**DOI:** 10.14740/jocmr2048w

**Published:** 2014-12-29

**Authors:** Dinu Vermesan, Ilie Trocan, Radu Prejbeanu, Dan V Poenaru, Horia Haragus, Damian Gratian, Massimo Marrelli, Francesco Inchingolo, Monica Caprio, Raffaele Cagiano, Marco Tatullo

**Affiliations:** a Department of Orthopedics and Trauma, University of Medicine and Pharmacy “Victor Babes”, Timisoara, Romania; b Vest University “Vasile Goldis”, Arad, Romania; c Calabrodental Clinic, Maxillofacial Unit, Crotone, Italy; d Marrelli Hospital, Orthopedics and Traumatology Unit, Crotone, Italy; e Department of Interdisciplinary Medicine, Medical Faculty, University of Bari “Aldo Moro”, Italy; f Department of Biomedical Sciences and Human Oncology, Medical Faculty, University of Bari “Aldo Moro”, Italy; g Tecnologica Research Institute, Biomedical Section, Crotone, Italy; h These authors contributed equally to this research paper.

**Keywords:** Incomplete tears, Meniscus *in situ*, Anatomic single bundle, Anterior cruciate ligament reconstruction

## Abstract

**Background:**

There is no consensus regarding the use of retaining or replacing cruciate implants for patients with limited deformity who undergo a total knee replacement. Scope of this paper is to evaluate whether a cruciate sparing total knee replacement could have a reduced operating time compared to a posterior stabilized implant.

**Methods:**

For this purpose, we performed a randomized study on 50 subjects. All procedures were performed by a single surgeon in the same conditions to minimize bias and only knees with a less than 20 varus deviation and/or maximum 15° fixed flexion contracture were included.

**Results:**

Surgery time was significantly shorter with the cruciate retaining implant (P = 0.0037). The mean duration for the Vanguard implant was 68.9 (14.7) and for the NexGen II Legacy was 80.2 (11.3). A higher range of motion, but no significant Knee Society Scores at 6 months follow-up, was used as controls.

**Conclusions:**

In conclusion, both implants had the potential to assure great outcomes. However, if a decision has to be made, choosing a cruciate retaining procedure could significantly reduce the surgical time. When performed under tourniquet, this gain does not lead to reduced blood loss.

## Introduction

Total knee replacement is one of the most successful currently performed arthroplasty. National registries predict a constant rise in the number of their annual procedures. There is still a constant debate regarding the use of cruciate retaining or replacing implants in patients with limited deformity. While there are subtle different benefits for each type, the overall result has always been proved to be the same. This has been previously studied by comparing the cruciate retaining and replacing implant of the same system with regard to functional outcome AMK [1], NexGen [2], Genesis II [3] and range of motion (ROM) [4, 5].

All conclude that, at a minimum of 2 years follow-up, functional scores (Knee Society Score (KSS), Western Ontario and McMaster Universities Arthritis Index (WOMAC), and SF-36) were similar but with a clinical not significant increase in ROM with the posterior replacing design. These findings are maintained even when comparisons were made in bilateral knees [6], mobile bearing [7] and high flexion implant designs [8]. The most common presentation, for knee arthroplasty, is a patient with limited varus deformity, generated by the higher prevalence of arthritis in the medial compartment. The relatively limited coronal plane deviation at surgery is determined by the accompanying intense clinical symptoms and high availability of the procedure. Another perspective is the high strain on the current health care systems worldwide which emphasizes better management and efficient use of resources. The posterior cruciate ligament replacing implants offer a more predictable procedure and outcomes, in comparison to their preservation. Nevertheless, this comes with a more invasive resection on the femur and, sometimes, with a more laborious procedure. We therefore aimed to investigate whether, or not, a cruciate retaining total knee replacement could be a more efficient approach for both provider and patient in most cases. The primary objective of our study was to evaluate if there is a significant difference in operating time between the two procedures with equivalent outcomes. The secondary objective was to determine if this also leads to a reduced perioperative blood loss with the cruciate retaining implant.

## Material and Methods

For this purpose, we performed a randomized study on 50 subjects consecutively enrolled over 16 months in a University Hospital. All surgical procedures were performed by a single experienced knee surgeon in the same operating room and assisted by the same team in order to minimize variables. Both males and females were considered eligible if they were older than 55 years, could comply with the postoperative follow-up visits and had a varus deviation of less than 20 and/or fixed flexion contracture of maximum 15°. Involved subjects have given their informed consent and this study protocol has been approved by the University of Medicine and Pharmacy “Victor Babes” Timisoara Committee on human research.

Preoperative planning was done using standard AP and lateral non-weight bearing radiographs. When in doubt, either skyline axial view of the patella, AP weight-bearing or whole leg CT scanograms were added as needed. Both primary and inflammatory diseases were included. Patients with previous contralateral knee replacement were also deemed eligible. A clinical significant lower limb deformity, posttraumatic or valgus knee arthritis or any general condition (neurologic, and inflammatory) which interfered with the patient’s potential ability to ambulate unassisted, were conditions for exclusion. Patient demographics are summarized in Table 1.

**Table 1:** Descriptive Statistics of the Patient’s Data at the Point of Index Surgery

N	M/F	Age (SD)	BMI (SD)
CR = 25	15/10	68.8 (6.9)	32.6 (7.1)
PS = 25	22/3	68.4 (6.3)	33.4 (7.5)

M/F: male and female number; SD: standard deviation of the mean for 95% confidence interval; BMI: body mass index.

All procedures used either cruciate sparing Vanguard^®^ CR (Biomet) or cruciate replacing NexGen^®^ Legacy II PS (Zimmer) implants. The femoral osteotomies were performed using intramedullary guidance and posterior (condyle) referencing. The box cuts for the posterior stabilized implant were done without a reciprocal saw. A default setting of 6° of valgus and 3° of external rotation for the femoral component were used for both implants. The tibial cut used extramedullary guides. For the cruciate retaining implants, a posterior slope resembling local anatomy, allowed a proper gap balancing determined by using a preoperative lateral X-ray and an intraoperative inclination of the lateral plateau [9]. For the posterior stabilized implant, we aimed for a neutral slope to the tibial axis. The implants were cemented in a single stage. The patella was never resurfaced. Five instrument trays were used with the cruciate sparing implant in comparison to seven for the cruciate replacing. The same medial para-patellar approach was used for all cases with eversion of the patella. All surgeries were performed with transient ischemia by use of standard 400 mm Hg band pressure. The band was inflated before skin incision after a minimum of 3 min of postural drainage. It was deflated after wound closure using three layers of non-absorbable interrupted sutures and compressive (elastic bandage) dressing. The drain was clamped for the first 2 h. All patients underwent the same postoperative physical therapy regimen started on the second day. Thromboprophylaxis was started on the evening of the surgical session and continued for 28 days using a single daily injection of low molecular heparin [10]. Perioperative bleeding was measured as hemoglobin dropping on the surgical day (morning to evening). Based on a retrospective analysis of the tourniquet times for both procedures, it was estimated 15 min standard deviation. We therefore computed a sample size of 25 knees for each group (80% power) and a significance level less than 0.05 (two-tailed *t*-test). A predetermined randomization list allowed us to perform similar preoperative planning for all cases. Continuous data were analyzed using descriptive statistics and unpaired *t*-test to compare the two means. Data processing was performed using GraphPad Prism 6 (GraphPad Software, Inc.). The study was approved by our hospital ethics committee and all measures were taken to ensure patients for safety and privacy, including intervention if one of the techniques proved unequivocal superiority. Clinical evaluation was made using the KSS [11].

Together with the WOMAC and ROM, these are the most widely used outcomes to compare cruciate sparing and replacing implants [12]. A single 6 months follow-up visit was used to determine similarity of final outcome for both implants, since these was not an objective of our study, but only a control measure; the 6 months results have shown to be predictive of the final (2 years) outcome [4, 5]. An unofficial local translation of the scores was administered at 1 year postoperatively in order to determine the outcome. All authors agreed on a local translation of the original KSS scale as described by Likert-scale patient questionnaires forms (www.orthopaedicscore.com) for a theoretical floor of 0 and ceiling of 100. The patients were instructed to answer all questions and to complete the forms with assistance (when needed) from one of the authors. All ROM measurements were evaluated in supine position as maximal passive flexion quantified with a hand held goniometer.

## Results

**Figure 1:**
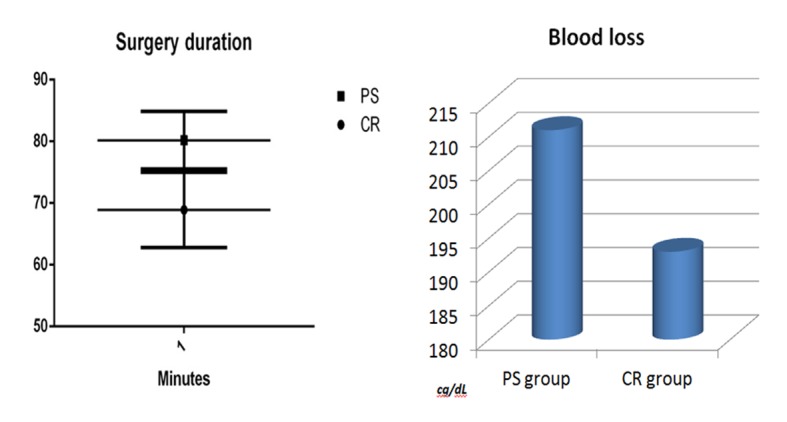
Chart distribution of operating time comparison.

The surgery time was significantly shorter with the cruciate retaining implant (P < 0.005). The mean duration for the Vanguard implant was 68.9 (14.7) and for the NexGen II Legacy was 80.2 (11.3). The perioperative drop in hemoglobin was not significantly (P = 0.4948) higher in the PS group: 2.11 (0.94) compared to 1.93 (0.91) g/dL. This was also the case with regard to blood transfusion: four cases in the PS and three in the CR group received one unit of red blood cells (Fig. 1). All patients returned for the 6 months follow-up (5 - 7 months). The collected data are scheduled in Table 2.

**Table 2:** Data Collected at 6 Months Follow-Up

N	KSS (SD)	ROM (SD)
CR = 25	83.4 (8.5)	100 (10)
PS = 25	86.1 (5.7)	110 (15)
P	0.1934	0.0079

KSS: Knee Society Score average of the means for symptoms and function; ROM: range of motion; SD: standard deviation of the mean for 95% confidence interval.

Patients from both groups had results which could be considered favorable when compared to expected follow-up after such a procedure. After 6 months, a follow-up was maintained in the same manner as for the rest of the patients. Survivorship and long-term outcome are still being followed on a yearly basis.

One case, in the required re-intervention during this period for wound infection, has been solved with debridement, aspirative drainage and antibiotic therapy.

Three cases, in the CR group, and one in the PS group, evidenced stiff knees at 6 months (ROM less than 80°).

## Discussion

In our study group, the cruciate retaining implant had a clear advantage over the posterior stabilized, with respect to operating time. Our data come from a single surgeon cohort, using the same technique for all cases, as they offered a consistent uniformity of comparisons together with minimized bias. The main author had consistent experience with both implants. To our knowledge, this is the only report focusing on the duration of surgery and possible benefit to blood loss between CR and PS total knee replacements. The most important purpose of our study is to compare the two used systems which consequently involve also the instrumentation and surgical technique used. Looking forward, it is also possible to improve surgical time and lead to a more efficient management with comparative results between different total knee systems.

When we look at literature reviews, there is a unanimously finding of better postoperative ROM in favor of posterior cruciate replacement. However, this is generally reported as around 5° and no differences in the KSS, WOMAC, component alignment, tibial posterior slope, joint line and incidence of complications [13]. This leads us to conclude that the reported gain in amplitude of movement has no effects on the clinical outcome, knee function or patient satisfaction, which are the most important parameters for any arthroplasty procedure. Similar outcomes, with reduced ROM, have led to studies evaluating comparative biomechanics of the two procedures. There are differences that probably contribute together for final results. Retention of the posterior cruciate ligament does not appear to significantly improve proprioception and balance [14]. In addition, Victor et al found that femoral roll-back was greater and more consistent with cruciate-substituting and this was predictive of the maximum flexion [15]. In contrast, a different study observed that posterior cruciate-substituting only showed a significant increase in posterior femoral translation after 90° [16]. Unconstrained implants are associated with less joint line changes [17], are superior in achieving equalized rectangular extension and flexion gaps [18] and can even produce improved survival [19]. By comparison, posterior stabilized knees are less susceptible to subtle changes such as the magnitude of posterior condylar offset which can correlate with a postoperative change in flexion in cruciate retaining systems [20, 21]. Bell et al found that intraoperative tourniquet release was associated with a significantly lower reduction in hemoglobin with respect to its release after wound closure [22]. In addition, a recent meta-analysis found that tourniquet release after wound closure could shorten the operation time but may not reduce the blood loss [23]. Our hypothesis was that, in similar conditions, a reduced surgical time might also reduce perioperative blood loss. This was not found to be significant, mainly because the operative gain was under transient ischemia and postoperative hemorrhage weight more towards total loss. In addition, the drop in hemoglobin was not adjusted for dilution.

### Conclusions

We conclude that both implants have the potential to assure great outcomes. However, if a decision has to me made, choosing a cruciate retaining procedure can significantly reduce the surgical time. When performed under tourniquet, this gain does not lead to reduced blood loss.
